# Penicillin Kills *Chlamydia* following the Fusion of Bacteria with Lysosomes and Prevents Genital Inflammatory Lesions in *C. muridarum*-Infected Mice

**DOI:** 10.1371/journal.pone.0083511

**Published:** 2013-12-23

**Authors:** Maud Dumoux, Sylvain M. Le Gall, Mohamed Habbeddine, Christiane Delarbre, Richard D. Hayward, Colette Kanellopoulos-Langevin, Philippe Verbeke

**Affiliations:** 1 Laboratory of Inflammation, Gestation and Autoimmunity, Institut Jacques Monod-UMR 7592, CNRS and University Paris Diderot, Paris, France; 2 Institute of Structural and Molecular Biology, University College London & Birkbeck, University of London, London, United Kingdom; Midwestern University, United States of America

## Abstract

The obligate intracellular bacterium *Chlamydia* exists as two distinct forms. Elementary bodies (EBs) are infectious and extra-cellular, whereas reticulate bodies (RBs) replicate within a specialized intracellular compartment termed an ‘inclusion’. Alternative persistent intra-cellular forms can be induced in culture by diverse stimuli such as IFNγ or adenosine/EHNA. They do not grow or divide but revive upon withdrawal of the stimulus and are implicated in several widespread human diseases through ill-defined *in vivo* mechanisms. β-lactam antibiotics have also been claimed to induce persistence *in vitro.* The present report shows that upon penicillin G (pG) treatment, inclusions grow as fast as those in infected control cells. After removal of pG, *Chlamydia* do not revert to RBs. These effects are independent of host cell type, serovar, biovar and species of *Chlamydia*. Time-course experiments demonstrated that only RBs were susceptible to pG. pG-treated bacteria lost their control over host cell apoptotic pathways and no longer expressed pre-16S rRNA, in contrast to persistent bacteria induced with adenosine/EHNA. Confocal and live-video microscopy showed that bacteria within the inclusion fused with lysosomal compartments in pG-treated cells. That leads to recruitment of cathepsin D as early as 3 h post pG treatment, an event preceding bacterial death by several hours. These data demonstrate that pG treatment of cultured cells infected with *Chlamydia* results in the degradation of the bacteria. In addition we show that pG is significantly more efficient than doxycycline at preventing genital inflammatory lesions in *C. muridarum*-C57Bl/6 infected mice. These *in vivo* results support the physiological relevance of our findings and their potential therapeutic applications.

## Introduction

Obligate intracellular bacteria of the genus *Chlamydiaceae* are the causal agents of a spectrum of human diseases of public health importance. *Chlamydia trachomatis (C.t)* infections remain the most frequent sexually transmitted bacterial disease, and the leading cause of infectious blindness (trachoma) worldwide [1], [2].

Infection by *Chlamydiaceae* follows a unique developmental cycle, lasting 30–100 hours, depending on the strain and species. Host cell infection is caused by a small, infectious yet metabolically inert form, the elementary body (EB, 0.3 µm diameter). Upon internalization, EBs differentiate into a larger, metabolically active form, the ‘reticulate body’ (RB, 1 µm). RBs forge a replicative niche within a membrane-bound compartment, termed an ‘inclusion’, by hijacking key eukaryotic processes. This includes blocking cellular apoptosis and the fusion of lysosomes with the inclusion [3]–[7]. RBs subsequently replicate by binary fission, prior to re-differentiating into EBs, which are released to re-infect neighboring cells [8].

Primary chlamydial infections are generally cleared by the host innate and adaptive immune responses. However, when chronic infection persists, local inflammatory responses can cause fibrosis and mucosal scarring and lead to severe sequelae in ocular tissue including blindness, and in the genital tract, including infertility, ectopic pregnancy and various gestation pathologies. Recent studies (reviewed in [2], [9]) argue that chronic infections are caused by an atypical intracellular form of the bacteria termed a ‘persistent’ form (PF). PFs have a low metabolic activity and are suspected to reside in tissues for years. They are non-infectious and hence cannot grow *ex vivo*. Several different host cell culture models have been developed to study PFs, after *in vitro* induction of persistence [9], [10]. Physiologically, persistence may be induced by locally high levels of IFNγ produced in response to infection, or by adenosine, released after lysis of infected neighboring cells. Consistent with this, the chlamydial developmental cycle can indeed be manipulated by adenosine in cell culture [11]. It is generally agreed that a persistent state is characterized by inclusion growth arrest and development from RBs of few non-dividing, non-infectious and slightly enlarged (∼ 2 µm in diameter) PFs. PFs can be reactivated and become infectious when the persistence inducer is removed, a common feature in all classical persistence models [2], [9], [10].

β-lactam antibiotics have also been claimed to induce chlamydial persistence in cultured cells [2], [9], [10]. Strikingly, while *Chlamydiaceae* are susceptible to this class of antibiotic, the recognized target, peptidoglycan, is absent: this feature is known as the “*Chlamydia* paradox” [12]. In the 1970s, penicillin G (pG) treatment of *Chlamydia*-infected cells was shown to affect RB division and induce apparent PFs [13]–[20]. Although current literature favors the hypothesis that β-lactam antibiotics induce *Chlamydiaceae* to persist *in vitro* and *in vivo,* older observations suggested that inclusion and bacterial morphologies following pG treatment differed from archetypal PFs, and that pG-treated *Chlamydiaceae* could not become infectious again after the withdrawal of pG [15], [21], [22].

Our work shows that pG treatment does not affect the growth kinetics of the inclusion, a hallmark of classical persistence inducers, but triggers the formation of much larger (5–10 µm) abnormal bacterial forms. Moreover, we demonstrate that pG withdrawal from *Chlamydia*-infected cultures does not restore infectivity. We show that pG treatment causes chlamydial death in multiple host cell types by inducing the entry of host lysosomes into the bacterial inclusion. Finally, our *in vivo* experiments show that mice vaginally-infected with *C. muridarum,* and treated per os by pG or doxycycline, an antibiotic of the Tetracycline family, eliminate the bacterial load in the lower genital tract much faster than untreated infected mice. However, only pG-treated mice are significantly protected from severe genital inflammatory lesions of the genital tract, when compared to untreated infected mice or infected mice treated with doxycycline. These data support the physiological relevance of our *in vitro* observations. They also suggest that pG-treatment of human *Chlamydia* genital infections could help reduce inflammatory lesions in the upper genital tract.

## Materials and Methods

### Ethics Statement

Six week-old C57Bl/6 female mice were housed in specific pathogen-free conditions and used according to European and NIH institutional guidelines. The use of mice in the manuscript was approved by the local Institutional Animal Care and Use (IACUC) and ethics Committee, officially referred to as "Animal Experimentation Ethical Committee, Buffon" (CEEA-40). The approved protocol number is CEB-05-2012. The current version of this protocol is valid until March 29 2015. Vaginal infection was performed under pentobarbital anesthesia, and all efforts were made to minimize suffering or discomfort.

### Bacterial strains, Cultures and reagents

All cell types (HeLa, RL-95-2, THP-1) were obtained from and cultured as recommended by ATCC (Manassas, VA), in 75 cm^2^ tissue culture flasks for maintenance and in 12- or 24-well plates containing coverslips, or in Lab-Tek chamber slides, when appropriate. THP-1 cells were differentiated into macrophages using PMA overnight at 0.25 µM in culture medium. *C. trachomatis* serovar L2 was from ATCC, *C. trachomatis* serovar D was kindly provided by Dr. De Barbeyrac (University of Bordeaux II, Bordeaux, France) and *C. muridarum* was a gift from Dr. Roger Rank (University of Arkansas, Little Rock, Arkansas, United States). Bacteria were routinely propagated in HeLa cells as previously described and stored at –80°C in sucrose-phosphate-glutamic acid (SPG) buffer (10 mM sodium phosphate [8 mM Na2HPO4–2 mM NaH2PO4], 220 mM sucrose, 0.50 mM Lglutamic acid) for later use [23]. The number of bacterial inclusion forming units (IFU) was determined using a previously described method [24]. Culture media (DMEM, HAMF F12, RPMI-1640), fetal calf serum and gentamycin were purchased from Invitrogen (Carlsbad, CA, USA). Cycloheximide (CHX), staurosporine (STS), adenosine (Ad), erythro-9-(2-Hydroxy-3-nonyl)adenine (EHNA), penicillin G (pG) and doxycycline (Dox) were from Sigma-Aldrich (St Louis, MO, USA). Lysotracker, Hoescht and pepstatin-FL-BODIPY were from Invitrogen (Carlsbad, CA, USA). Anti-*Chlamydia* genus-FITC antibody was from Argene (Argen Biosoft 12-114, Varhilles, France), anti-cathepsin D from Santa Cruz (Santa Cruz Biotechnology, CA, USA) and secondary antibody from Beckman Coulter (Fullerton, CA, USA).

### Chlamydial infection of cultured cells and treatment with pG

To examine the effects of pG on chlamydial infection, cells were cultured up to 70% confluence in culture medium containing no antibiotic and no CHX (infection medium) and were infected either with *C. trachomatis* or *C. muridarum* at an inclusion forming unit (IFU) of 1. Infections were performed at 37°C for 90 min, then cells were washed to eliminate dead bacteria. At different times post infection (hpi), culture medium was replaced by infection medium or medium containing different concentrations of pG (.01 to 100 IU/mL). At different times after infection and pG treatment, cultures were either fixed in 4% neutral-buffered paraformaldehyde for 30 min to be processed for microscopy, or harvested for infectivity measurements of the progeny (titration assay) as previously described [23].

### Induction of chlamydial persistence with adenosine/EHNA treatment

Persistence of the L2 strain of *C.trachomatis* was induced as previously described [11] with minor modifications. Briefly, HeLa cells were infected with *C. trachomatis serovar* L2 at an IFU of 0.5. At 1 hpi, Ad (400 µM) was added to the culture medium and replaced every hour from 1 to 8 hpi. Then, medium containing both Ad (400 µM) and EHNA (25 µM) was added to prevent adenosine degradation. This medium was replaced at 24 hpi and every 8 h thereafter to maintain the persistent phenotype.

### Reversion assay

HeLa monolayers grown on coverslips were infected with *C. trachomatis* serovar L2 and treated with either different concentrations of pG or with Ad/EHNA as described. At 24 hpi or 32 hpi, the culture was extensively washed and medium was replaced by infection medium (reversion step). At 75 hpi, the cell layer and culture medium were collected and a titration assay performed as previously described [23]. To examine whether inhibition of host cell protein synthesis could allow the recovery of infectivity of the progeny from pG-treated cells, HeLa cells were treated with CHX at 1 µg/mL 4 h prior to infection. From 3 hpi to 24 hpi, pG (100 IU/mL) was added to the culture medium containing CHX or not. At 24 hpi, pG was removed, and culture was continued up to 100 hpi in the presence or absence of CHX, before titration assays were performed.

### Preparation of RNA and analysis of gene expression

10^5^ HeLa cells were seeded prior to infection and incubation with either pG or Ad/EHNA. At 75 hpi, total RNA was isolated using an RNeasy Mini Plus Kit (Qiagen, Hilden, Germany). DNase I treatment was performed (1 h at 37°C). RNA was reverse-transcribed using random hexamer primers and an affinity Script MT reverse transcriptase (Agilent, Santa Clara, CA), according to the manufacturer’s instructions. Control PCR reactions were performed using isolated RNA to verify the absence of genomic DNA contamination. PCR was processed using Taq Polymerase (Invitrogen, Carlsbad, CA, USA) and primers designed for bacterial pre-16S rRNA (Fw: gCCAgTATAgATgCTTgTgAggA Rv: CTgCAgCCTCCgTAgAgTCTgggCAgTgTC) and eukaryotic GAPDH rRNA (Fw: gAgTCAACggATTTggTCgT Rv: TTgATTTTggAgggATCTCg) [25]. Semi-quantification of the resulting PCR products was achieved by agarose gel electrophoresis and UV detection.

### Sensitivity to apoptosis

HeLa cells were infected with *C. trachomatis* serovar L2 and treated with pG at either 2 hpi or 29 hpi. Cells were incubated with 1 µM of STS at 31 hpi, fixed at 40 hpi and stained with Hoechst and a FITC-conjugated anti-*Chlamydia* antibody. More than 10 microscopy fields were scored for each condition in triplicate. Under these conditions, STS treatment induces apoptosis in 90–100% of uninfected cells. Percentages of infected cells containing apoptotic nuclei were calculated.

### Confocal microscopy

Cells were cultured on coverslips and infected with *Chlamydia*. When appropriate, cell layers were treated with pG at 3 hpi. Cells were fixed at 24 hpi, permeabilized and incubated (2 h, RT) with an anti-cathepsin D antibody diluted in PBS containing 1% (w/v) BSA. After extensive washing, cells were stained for 1h at RT with a Texas red-conjugated goat anti-rabbit IgG secondary antibody, with a FITC-conjugated anti-*Chlamydia* antibody and then counterstained for 5 min with Hoechst. Isotype controls were used in all experiments. Coverslips were mounted and observed using a confocal microscope (Leica, TCS Sp5 AOBS tandem, Leica, Mannheim, Germany) equipped with two diode lasers (1 and 25 nW) emitting at 405 nm and 561 nm, respectively, and an argon laser (100 nW) emitting at 488 nm. Emission signals were recorded at 411–481 nm for Hoechst, 493–555 nm for FITC and 591–703 nm for Texas Red. Each experiment was performed, acquired and analysed similarly, and repeated at least three times.

### Labeling of cultured cells with lysotracker-red

Cells cultured on glass-bottomed Lab∼Tek chamber slides (Thermo Fisher Scientific, Roskilde, DK) were infected and treated with pG at 3 hpi. At 22 hpi, cell layers were incubated with 10 µM of lysotracker-red diluted in DMEM (30 min, 37°C). Medium was replaced by infection medium and slides were placed in an environmental chamber at 37°C with 5% CO_2_ and observed in time lapse videomicroscopy using a Leica confocal microscope equipped with a tandem resonant scanner. Excitation was performed at 561 nm using a diode laser (1 nW) and emission signal was collected between 565 and 641 nm. Images were acquired every 2 min for up to 4 h.

### Cathepsin D-active site staining

The active sites of cathepsin D have been stained using the fluorescent Bodipy-pepstatin-FL probe (Invitrogen, Carlsbad, CA, USA) [26]. Briefly, infected cells treated or not with pG were fixed at 32 hpi, permeabilized and washed with sodium acetate buffer (pH 4.5). Coverslips were incubated for 1 h at RT with 5 µM of Bodipy-pepstatin FL diluted in sodium acetate buffer. After extensive washes in Tween-acetate buffer, coverslips were mounted and observed by fluorescence microscopy.

### Mouse genital tract infection and antibiotic treatments

Six week-old C57Bl/6 female mice were purchased from Charles River Laboratories (France). Animals were randomly divided into 4 groups: non-infected (n = 10), infected non-treated (n = 35), infected + Dox (n = 29), infected + pG (n = 36). Four days prior to infection, animals received 2.5 mg of progesterone subcutaneously (Depo-Provera, Pfizer, France) to synchronize their estrus cycle. Mice to be infected were anesthetized by intra-peritoneal injection of 24 mg of pentobarbitone per kg of body weight and were inoculated intra-vaginally with 3×10^6^ IFUs of live *C. muridarum* organisms in 20 µl of SPG buffer. Non-infected animals received the same volume of SPG. From 10 up to 30 days after infection, 2 groups of mice received either Dox or pG, daily at 10 mg/ml in drinking water supplemented with 7.5% sucrose. Control infected non-treated mice were given drinking water supplemented with 7.5% sucrose only. The amount of circulating antibiotic was determined for each group by measuring the daily volume drunk by mice (daily weighing of the bottles) and using the factors of intestinal absorption and excretion for each molecule. Depending on the experiment, we calculated that mice treated with Dox received from 6.55 to 11.42 µmol/animal/day and mice treated with pG received from 3.65 to 8.72 µmol/animal/day.

### Monitoring vaginal Chlamydia live infection

For the three groups of infected animals (infected non-treated (n = 5), infected + Dox (n = 5), infected + pG (n = 6)), the vaginal bacterial load was monitored by collecting vaginal swabs every 2 or 3 days after intra-vaginal bacterial infection, until 32 days post-infection. Chlamydia organisms were recovered from swabs and triplicates of serially diluted samples were used to infect HeLa cells grown on 96 well plates. After 24 h in the presence of CHX (1 µg/mL), cells were fixed using PFA 4% and stained using May-Grünwald Giemsa dyes. At least four random fields were counted per well. Wells showing cytotoxicity were excluded. The total number of IFU per swab was calculated based on the percentage of infected cells, the total number of cells grown in each well, the number of fields counted per coverslip, the average of triplicates, the volume and the dilution factors of the recovered samples. Average and standard deviation of total number of IFU/swab was calculated for each group and each time point and converted into log_10._

### Scoring of Uterine lesions

Mice from each group were sacrificed 90 days after infection and the genital tract was isolated for pathology examination. Both incidence and severity of fluid-filled cysts along the uterine horns were recorded for each mouse and compared between different groups. The severity of those cysts was scored based on their size. Absence of lesion was scored as 0; cysts visible only with binocular amplification were scored as 1; cysts smaller than 3 mm were scored as 2; cysts larger than 3 mm were scored as 3.

### Histopathology

Immediately after the evaluation of macroscopic uterine lesions, the uterine horns were fixed in 4% buffered paraformaldehyde, and embedded in paraffin. Transversal 5-µm sections were performed in the middle part of the horns, stained with Hematoxylin and Eosin, and observed at a 40 X magnification. Changes in morphology of the uterine horns and the presence of inflammatory cell infiltrates were monitored.

### Statistical analysis

Data are presented as the mean ± standard deviation of “n” experiments, and p values were calculated using a two-tailed two-sample equal variance Student’s t test. For qualitative data analyses, the non-parametric Mann-Whitney test was used. Statistically significant differences are noted as follows: (* p<0.05), (** p<0.01), (*** p<0.001).

## Results

### pG induces abnormal bacterial forms in *Chlamydia-*infected cells

Treatment of infected cell cultures with pG, or adenosine associated with EHNA, has been reported to induce chlamydial persistence [1], [2], [11]. In the literature, persistence is defined by inclusion stasis, and reversion, *i.e.* the recovery of infectious activity following the removal of the persistence inducer.

**Figure 1:**
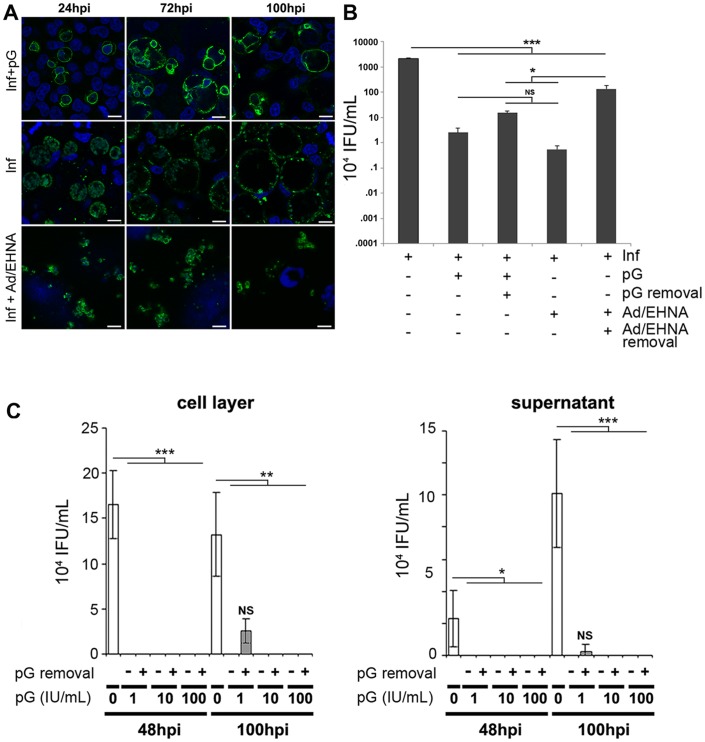
Penicillin G (pG) treatment *in vitro* does not induce classical persistence of *C. trachomatis*. HeLa cells infected by *C. trachomatis* serovar L2 were treated at 3 hpi with either pG (100 IU/mL) or Ad/EHNA, or left untreated. **A-** At different times after infection, cells were fixed and stained using an anti-*chlamydia* antibody (green) and Hoechst (blue). Scale bar: 10 µm. **B-** pG or Ad/EHNA were removed at 32 hpi from culture medium and culture was continued for 43 h. At 75 hpi, cells were processed for the titration of recovered infectious activity. **C-** HeLa cells infected by *C. trachomatis* serovar L2 were treated at 3 hpi either with 1, 10 or 100 IU/mL pG or left untreated. In some samples (+), pG was removed at 24 hpi from culture medium and culture was continued. At 48 hpi or 100 hpi, cell layers (left panel) and supernatants (right panel) were processed for the titration of recovered infectious activity. All experiments have been repeated at least 3 times. * correspond to p*<* 0.05, ** correspond to p <0.01, *** correspond to p < 0.001. See also Figure S1

To investigate the effects of pG further, we initially compared inclusion morphology during pG treatment to IFNγ or iron deprivation, the last two being assumed to be physiological inducers of persistence *in vivo*. However, neither IFNγ nor iron deprivation induced persistence in 100% of infected cells, hampering subsequent transcriptomic or biochemical analyses of persistent cells. Thus, we opted for the comparison of pG with adenosine (Ad)/EHNA treatment, which induced persistence in nearly all infected cells. The size of persistent bacterial inclusions remained constant for as long as one week (Figure 1A, after a 4 day treatment with Ad/EHNA). Within these inclusions, the average diameter of bacterial PFs was 2–3 µm. Both the static inclusions and the modest size of the PFs induced by Ad/EHNA treatment are characteristic of classical chlamydial persistence *in vitro*. In contrast to the bacterial inclusions produced in this persistence model (Ad/EHNA), inclusions produced in infected cells treated by pG are growing at the same rate as the inclusions developing in untreated cells, both lysing at 100 hpi. The diameter of abnormal bacteria (5–10 µm) induced by pG treatment was much larger than typical PFs. Both the inclusion growth and the size of bacterial forms following pG treatment strongly suggest that *Chlamydia* fail to enter a persistent state. We verified that the development of growing inclusions containing unusually large bacterial forms was independent of the host cell type or the serovar, biovar or species of *Chlamydia* (Figure S1A). Consequently, we conclude that unlike Ad/EHNA, pG treatment leads to the formation of very enlarged bacterial bodies, which we call “pG-forms”, contained within a growing inclusion, a phenotype inconsistent with classical persistence.

Another hallmark of classical persistence is the resumption of inclusion growth and the reemergence of infectious progeny following the withdrawal of the persistence inducer. Thus, the infectious capacity of bacteria after pG or Ad/EHNA removal was assessed (Figure 1B). A 75 hpi, we observed a significant bacterial growth (x250) when Ad/EHNA was withdrawn at 32 hpi. The growth was non-significant when pG was removed at the same time point (Figure 1B). To rule out any dose-dependent effect of the antibiotic and the possibility that infectious bacteria can be released from the host cells during pG-treatment, we performed two different experiments (Figure S1B and Figure 1C).

Using serial dilutions of pG, we showed that no infectious bacteria was observed in or out of the host cell at the dose of 1 IU/mL of pG (Figure 1C). This is also confirmed when pG was withdrawn at 48 hpi (Figure S1B). However, at concentrations lower than 0.1 IU/mL pG was less effective, most probably resulting from incomplete bactericidal efficiency and not from reversion (Figure S1B). This lack of reversion *in vitro* has also been observed for *C. muridarum*, down to 0.3 IU/mL (data not shown). This demonstrates that a second feature of classical chlamydial persistence, reversion upon removal of the persistence inducer, is also not observed following pG treatment.

Since our results appeared at odds with part of the literature claiming that pG induced persistence of *Chlamydiaceae*, we compared our experiments to previously reported protocols. Most of the groups that had reported reversion post pG-treatment had performed pG-treatment of infected cells in the presence of cycloheximide (CHX) to lower cell proliferation during infection. We performed reversion experiments using pG in the presence or absence of CHX. Surprisingly, these experiments showed that a partial recovery of infectivity was detected after pG withdrawal only in the presence of CHX, (Figure S1C). These conditions most likely account for the data discrepancies and suggest that *de novo* eukaryotic protein synthesis in the presence of pG prevents the recovery of infectious bacterial progeny following pG withdrawal.

### RBs are targeted by pG

**Figure 2:**
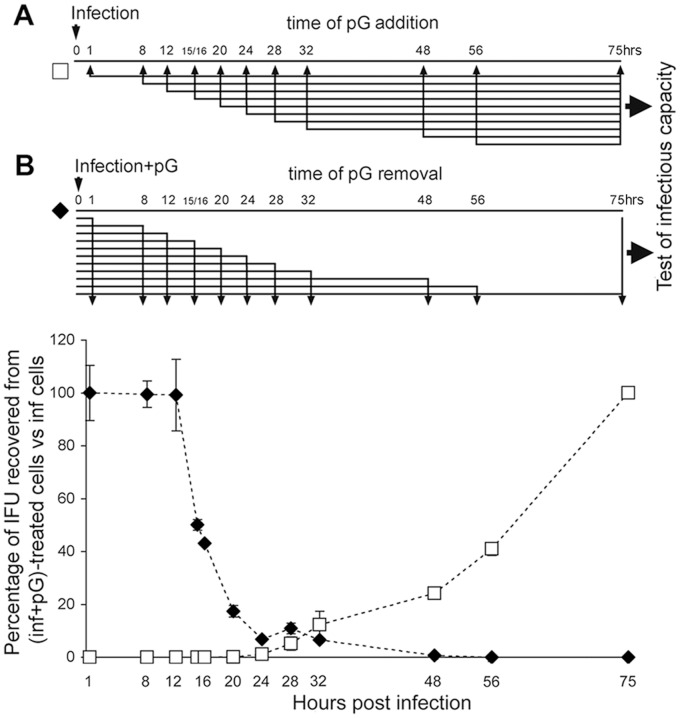
pG affects Chlamydia RB development. HeLa cells were infected by *C. trachomatis* serovar L2. Separate infected cultures were: **A-** treated with pG (100 IU/mL) from indicated times post-infection up to 75 hpi, before the titration of recovered infectious activity.(white squares); or **B-** treated with pG from 1 hpi until the indicated time. Cultures were continued after pG removal until 75 hpi, and then titrated for their recovered infectious activity (black diamonds). The infectivity potential was expressed relatively to non-treated infected cells. The experiments have been performed at least 3 times.

Next, we determined the window of pG action during the *C. trachomatis* cycle. When pG is added before 24 hpi and left in the culture, infectious bacteria were never recovered at 75 hpi (Figure 2, white squares). When pG is added at 24 hpi or later, a fraction of bacteria (increasing with hpi time) appear resistant to pG treatment as infectious progeny are detectable.

When the presence of pG in the culture medium does not exceed 12 hpi, the infectivity capacity of the progeny is not affected (Figure 2, black diamonds). By contrast, increasing efficacy against *C.trachomatis* is observed when the incubation with pG exceed 12hpi (to become maximal at 24–32 hpi) before removal. Altogether, these results indicate that chlamydial forms present in infected cells up to 12 hpi, as well as newly divided bacterial progeny present after 24 hpi, are not sensitive to pG. This strongly suggests that pG acts on RBs during their multiplication phase, whereas EBs appear insensitive to pG.

### pG treatment kills *Chlamydia*

**Figure 3:**
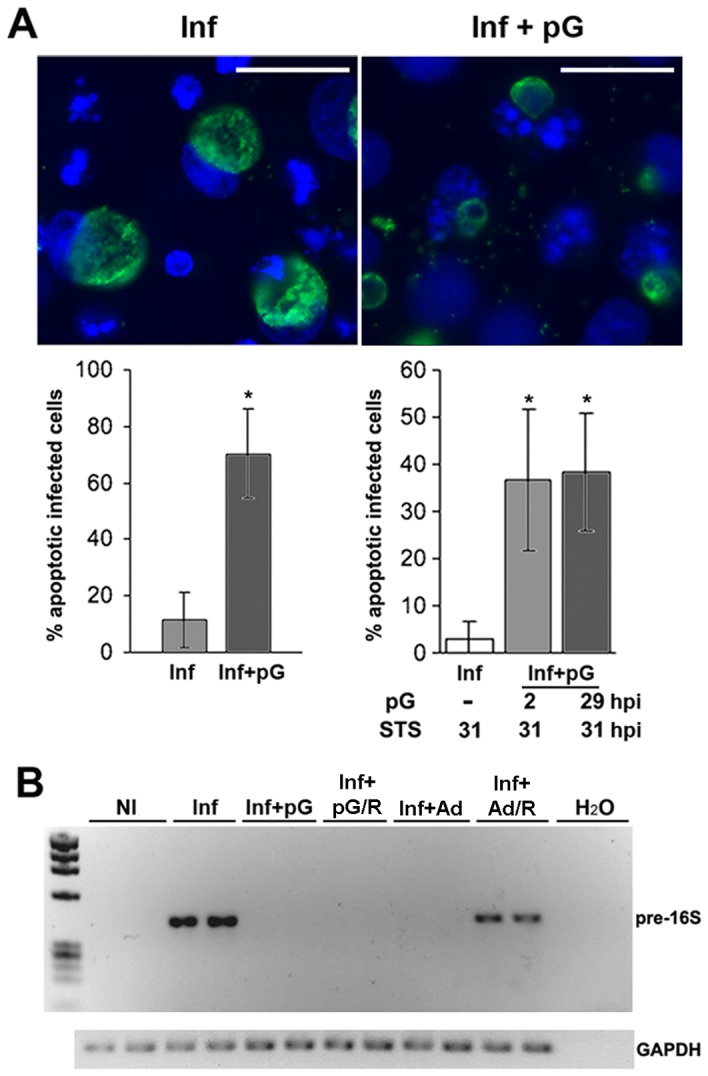
pG restores apoptotic pathways in the host cells and inhibits RNA biosynthesis of *Chlamydia*. **A-** HeLa cells were infected by *C. trachomatis* serovar L2 and treated with pG at 2 hpi or left untreated. At 21 hpi, STS was added to the culture medium. Cells were fixed at 48 hpi and stained as in Figure 1. Scale bar: 10 μm. Left panel: data representation from the pictures after calculation of the percentages of apoptotic infected cells. Right panel: Infected-HeLa cells were treated with pG at either 2 hpi or 29 hpi or left untreated. Then, STS was added at 31 hpi. Cells were fixed at 39 hpi and percentages of apoptotic infected cells were determined and presented as in the left panel. **B-** HeLa cells were infected by *C. trachomatis* serovar L2 and treated at 1 hpi with either pG or Ad/EHNA, or left untreated. In some experimental conditions (**R**), pG or Ad/EHNA were removed from culture medium at 32 hpi and the culture was continued for 43 h. At 75 hpi, cells were processed to measure pre-16S and GAPDH RNA expression. The experiments have been repeated 3 times. * correspond to p*<* 0.05.

Given these effects, we measured the impact of pG treatment of infected cells on the capacity of bacterial RBs to inhibit host cell apoptosis and to transcribe a housekeeping precursor of 16S rRNA, a classical key reporter of bacterial viability. When infected cells were treated with STS at 21 hpi, only 11.5±9.8% became apoptotic at 48 hpi while every uninfected control cell exhibited a typical apoptotic nuclear staining (Figure 3A). Under identical conditions, 70.4±15.9% of pG treated-infected cells were apoptotic. To evaluate the kinetics of this effect, infected cells were treated with pG at 2 hpi or 29 hpi, and subsequently incubated with STS at 31 hpi, and fixed 8h later. In both conditions, we observed the same percentage of infected and apoptotic cells (36.9±15% and 38.4±12.4%, respectively). These results demonstrate that pG has a rapid effect on the bacteria, since within a few hours, it causes an inhibition of the blocking of cellular apoptotic pathways due to infection.

We tested the expression of pre-16S rRNA at 75 hpi, after culture in different conditions: absence of drugs, presence of pG or Ad/EHNA from 3 to 75 hpi, and following their removal at 32 hpi in a reversion set up. During both treatments (Inf+pG and Inf+Ad), transcription of pre-16S RNA was below the threshold of detection (Figure 3B). However, upon withdrawal of these stimuli, only bacteria pretreated with Ad/EHNA resumed transcription of pre-16S rRNA.

The absence of newly synthesized pre-16S rRNA upon the removal of pG indicated that bacterial pG forms were no longer transcriptionally active. We chose to monitor pre-16S rRNA as it is critical for the bacterial survival and highly transcribed at a high turn-over rate. Thus, its absence strongly suggests that pG-forms are no longer viable.

### pG treatment induces the fusion of lysosomes with the pG-forms of *Chlamydia*

**Figure 4:**
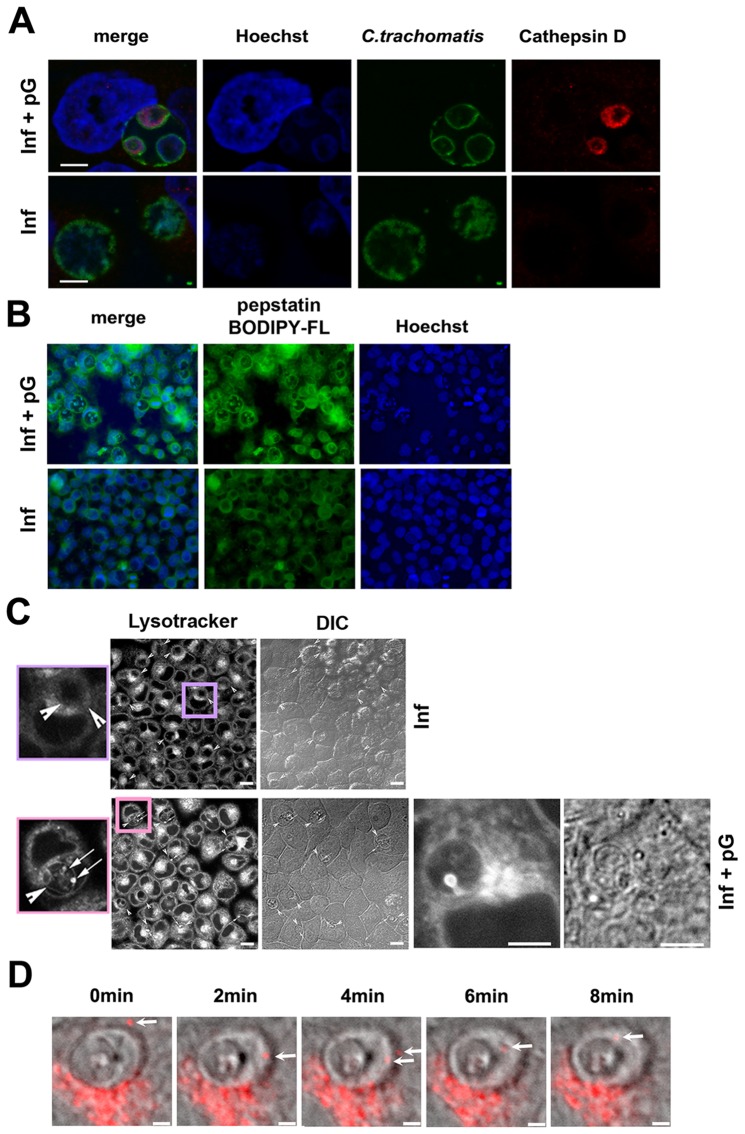
pG induces lysosome entry into bacterial inclusions. HeLa cells were infected by *C. trachomatis* serovar L2 and treated with pG at 3 hpi or left untreated. At 24 hpi, cells were either fixed and stained (A and B) or readily observed (C and D). Scale bar: 10 μm. The experiments have been repeated at least 3 times. **A-** Staining of pG-treated and untreated infected cells as in Figure 1, adding anti-Cathepsin D labelling (red). **B-** Staining of pG-treated and untreated infected cells using 5 µM BODIPY-pepstatin-FL (green) and Hoechst (blue). **C-** Cells were incubated with lysotracker for 30 min at 37°C before observation. Lysotracker localizes mainly in inclusions in pG-treated infected cells. Details show strong bright spots in these inclusions, suggesting a lysosome entry. Arrowhead: inclusion, arrow: bright spot. DIC: differential interference contrast. **D-** Time lapse using a confocal microscope with high resonance scanner. In grey, differential interference contrast, in red, lysotracker. A lysosome (white arrow) enters and stays inside an inclusion in pG-treated infected cells.

As pG-treated *Chlamydia* appeared non-viable and non-infectious, we examined how the bacteria were degraded, in particular whether inclusions might be targeted by lysosomes in pG-treated infected cells. Initially, acidic cellular compartments were tracked using cathepsin D staining. Inclusions in untreated cells excluded this marker while it accumulated in pG-forms (Figure 4A). This cathepsin D accumulation was not restricted to a particular bacterial strain or cell line, as equivalent results were obtained when endometrial RL95-2 cells were infected with *C.t* serovar L2 or Hela cells with *C.t* serovar D (Figure S2). Furthermore, we demonstrated that the recruited cathepsin D was enzymatically active using a fluorescent active site-specific probe BODIPY-pepstatin-FL (Figure 4B).

**Figure 5:**
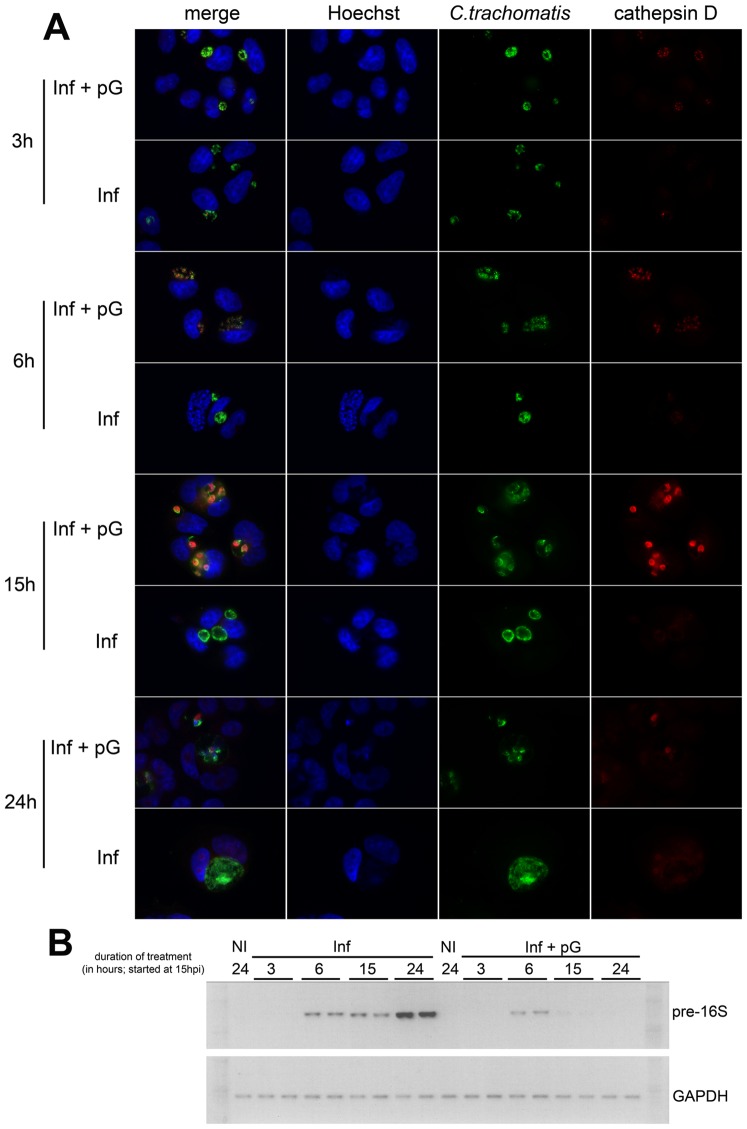
In the presence of pG, the recruitment of lysosomes precedes the death of *Chlamydia trachomatis*. HeLa cells were infected by *C. trachomatis* serovar L2. At 15 hpi, cells were treated or not with pG for 3 h (18 hpi), 6 h (21 hpi), 15 h (30 hpi), or 24 h (39 hpi). **A-** Cells were either fixed and stained as described in Figure 4 or **B-** processed to measure pre-16S and GAPDH RNA expression, as described in Figure 3. The experiments have been repeated 3 times.

To monitor the process of cathepsin D recruitment and activation, live labeling of lysosomes was performed using lysotracker red. This probe revealed an extensive membrane network, present only within the inclusion of pG-treated infected cells (Figure 4C). Surprisingly, bright lysotracker puncta were present within these inclusions (Figure 4C, inset), suggesting that intact lysosomes entered the inclusions. We used confocal videomicroscopy to study the dynamics of lysotracker-labelled compartments in pG-treated infected cells. Compartments loaded with lysotracker entered the inclusions and were recruited onto the membrane of pG-forms (Figure 4D and movie S1). Our data show that this unusual entry of lysotracker-positive compartments into the bacterial inclusion leads to the accumulation of active lysosomal protease cathepsin D within bacterial pG-forms. In addition, we showed that this recruitment was observed after only 3 hours of incubation with pG (Figure 5A). This gradual accumulation clearly preceded the decrease in pre-16S rRNA transcription (Figure 5B), which strongly suggests that these events are the cause of the bacterial death.

We sought to inhibit the fusion of lysosomes with the bacteria or the activation of cathepsin D by incubating pG-treated infected cells with NH_4_Cl or bafilomycin A1, prior and during infection and pG treatment. Ammonium chloride has been shown to inhibit the fusion of lysosomes with some cargo, while bafilomycin A1 specifically inhibits the H+-v-ATPase of the lysosome, regulating the pH of this organelle. None of these treatments prevented the accumulation of active cathepsin D in the pG-forms, nor avoided the irreversible loss of infectivity observed in pG-treated infected cells (data not shown). These results indicate that the fusion of lysosomes with the bacteria in pG-treated infected cells occurs via a pathway insensitive to NH_4_Cl. Moreover, the activation of cathepsin D in pG forms is independent of the activation of a lysosomal v-ATPAse.

### pG oral treatment of mice vaginally-infected with *C. muridarum* prevents the formation of uterine lesions

**Figure 6:**
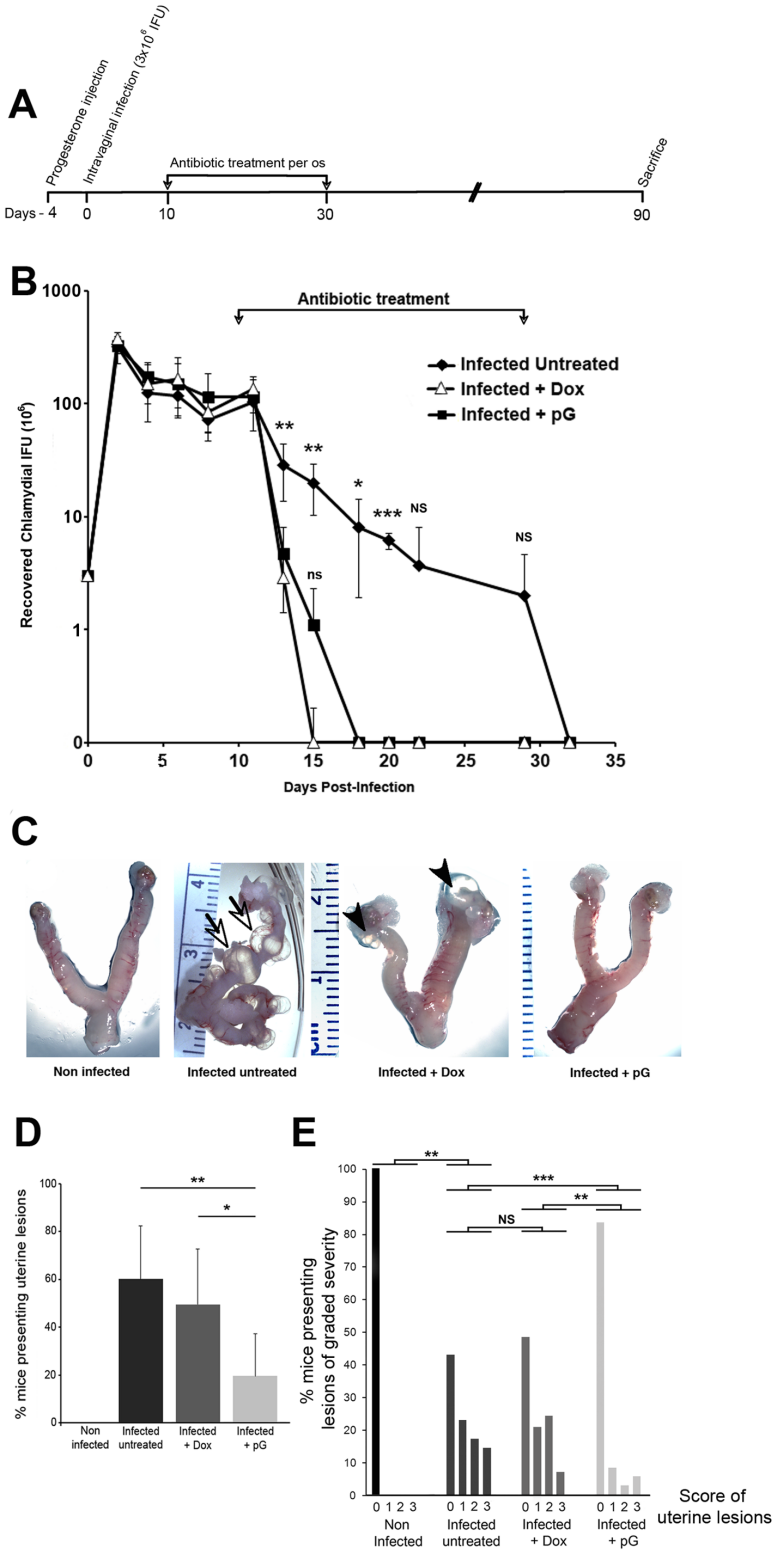
pG oral treatment prevents the development of uterine lesions in C57Bl/6 mice previously infected with *C. muridarum.* A- Female mice were synchronized in their cycle by a Progesterone injection and four days later, were infected intravaginally with 3×10^6^ IFUs of *C. muridarum*. After 10 days, animals were treated or not with 10 mg/mL of doxycycline or penicillin G for an additional 20 days. Mice were sacrificed at 90 days post infection. B- Infection course in infected mice either left untreated (black diamond), or treated *per os* with Dox (white triangle) or with pG (black square). Each data point represents the mean ± standard deviation of the number of inclusion forming units recovered from vaginal swabs collected from at least 5 mice per group. *: p< 0.05 compared to pG- and Dox-groups, **: p< 0.01 compared to pG- and Dox-groups, ***: p< 0.001 compared to pG- and Dox-groups. NS: non-significant compared to pG- and Dox-groups. ns: non-significant compared to Dox-group. C- Mice were infected with *C. muridarum* and treated as described in Figure 6A. Ninety days after infection, mice were sacrificed to collect and examine their entire genital tract. The pictures are representative of the cohorts studied. The presence of cysts is indicated by arrows, and hydrosalpinges by arrowheads. D- After sacrifice of the mice, the genital tract was isolated and the percentage of mice presenting uterine lesion in at least one horn was determined. Non-infected group (n = 10), infected non-treated group (n = 35), infected + Dox group (n = 29), infected + pG group (n = 36). * correspond to p*<* 0.05, ** correspond to p <0.01. E- After sacrifice, the severity of uterine lesion (cysts and hydrosalpinges) was scored as described in material and methods. The number of uterine horns without (0) or with varying degrees (1 to 3) of lesion severity was reported. The Mann Whitney test was used to compare the differences in lesion severity score between the different groups of mice, as indicated in the figure. NS: non-significant; ** correspond to p< 0.01; *** correspond t*o* p< 0.001.

In order to assess the physiological relevance of our *in vitro* observations, we decided to compare the effect of pG versus Doxycycline (Dox) treatment (or no treatment as control) in vaginally-infected mice. Four days after progesterone injection, groups of mice were infected intra-vaginally with *Chlamydia muridarum* (3×10^6^ IFUs). The infection was left to develop for 10 days before antibiotic treatments were given for 20 days (Figure 6A). Infected mice were treated with Dox or pG (10 mg/ml in drinking water with sucrose, in both cases) or left untreated (sucrose only in drinking water). The vaginal chlamydial shedding was monitored on the indicated days for a period of 32 days following bacterial challenge. As shown in Fig. 6B, untreated infected mice resolved the vaginal infection around 32 days. Mice infected and treated either by Dox or pG resolved the infection significantly earlier than the control group, respectively by day 15 and day 18 post-infection, 5 and 8 days after the start of treatments (no statistically significant difference between the two antibiotics). These results suggest that both antibiotic treatments increased early bacterial clearance following intravaginal *C. muridarum* challenge.

Ninety days after infection, mice were sacrificed for macroscopic examination of their genital tract to detect possible inflammatory lesions (fluid-filled cysts along uterine horns and hydrosalpinges). Results are presented in Figures 6C-E. The macroscopic examination of the uterine horns in the different groups of mice gave unequivocal results. Fifty seven percent of non-treated infected mice presented deformations of the horns with multiple fluid-filled cysts often associated with hydrosalpinges (Figures 6C-E). These anomalies were as frequent and as severe in Dox-treated animals than in infected non-treated mice (Figures 6D and 6E). In contrast, 83% of mice from the pG-treated group had no uterine lesions and 17% of the remaining mice developed less severe lesions than untreated mice or even mice treated with Dox (Figures 6D and 6E, statistically significant differences). We have performed histopathology analyses on transversal sections of paraffin-embedded uterine horns from mice in each group (Figure S3). Hematoxylin/eosin staining of sections revealed important anomalies in the non-treated infected group of mice compared to the control non-infected tissues. The lumen was very enlarged and distorted, the surrounding tissues were extended and all layers (both layers of myometrium and endometrium) had a much reduced thickness and were infiltrated by cells. Membranes surrounding liquid filled cysts were also present. Sections from Dox-treated infected mice showed an enlarged lumen and tissue distortions but to a lesser extent than untreated infected animals. In contrast, uterine horns from pG-treated infected mice did not present any detectable anomaly and were comparable to control uninfected uterine horns.

## Discussion

To the best of our knowledge, this is the first demonstration that pG-treatment of *Chlamydia*-infected cells *in vitro* reproducibly induces bacterial death.

Classical persistence has been defined *in vitro* on the basis of two main criteria: a non-developing inclusion containing few modestly enlarged, viable RBs [27], and reversibility, *i.e*. the capacity of PFs to resume a normal cycle after removal of the persistence inducer, and to produce an infectious progeny [2], [28], [29].

Although the present study was based on an *in vitro* model of pG-treatment of *C. trachomatis*-infected cells previously used by other groups, we have observed reproducibly that pG treatment allows a continuous growth of inclusions containing very few enlarged, noninfectious *Chlamydiaceae* forms. Other groups have reported similar findings [13], [15], [18]–[20], [30].

These observations, coupled with the fact that pG-forms of *C. trachomatis* cannot revert to infectious EBs after removal of pG even after 48 hpi, indicate that pG-forms differ from “classical” PFs.

Recently, Ouellete *et al.* have used very low doses of pG (8 IU/mL) to treat *C. trachomatis* serovar L2-infected HeLa cells [31]. In this study, they showed the development of large abnormal bacterial bodies similar to what we observe in our study. In both studies, even minute amounts of pG (0.08 IU/mL) reduce the infectious progeny by 4 logs. Ouellette *et al.* have tested different beta-lactams and have shown that pG is the most effective to reduce the infectious progeny from infected cell cultures. We confirmed this finding. Interestingly, when they removed pG from the culture medium at 24hpi, they recovered a small infectious activity, which is in agreement with what we have described in the present study. Indeed, we observed that when pG is removed from L2-infected cultures earlier than 48hpi, there is a significant probability that all initial EBs have not yet converted to RBs before the withdrawal of pG, and RBs only are sensitive to pG. Thus, some initial EBs could be converted to RBs after the removal of pG and then be able to multiply and produce new infectious EBs.

Finally, we have shown in our study that a brief (2 hrs) pG-treatment is sufficient to trigger an inhibition of chlamydial suppression of host cell apoptosis.

As it was hypothesized by Johnson and Hobson 35 years ago, but not demonstrated, the addition of pG soon after infection can inhibit bacterial virulence and allow the host cell to activate defense mechanisms, such as the targeting of lysosomes to the bacterial compartment [15].

We demonstrate here that pG treatment leads to the recruitment of active cathepsin D into pG-forms as early as 3 h post antibiotic treatment. Importantly, we observed the penetration of intact lysosomes into the atypical inclusion. The absence of fusion of lysosomes with the membrane of the inclusion is an unexpected finding, although the translocation of lipid droplets into inclusions has recently been described, suggesting a potential mechanism for the translocation of large intact bodies across the inclusion membrane [32]. The formation of this degradative environment leads to a complete lack of transcription of bacterial RNA such as the vital and highly transcribed pre-16S-rRNA. Another report showed that genes remain inactivated after withdrawal of pG from cultures of *C. pneumoniae*, suggesting that bacteria are dead following pG-treatment [22]. In contrast, the prevention of fusion of lysosomes with bacteria, and the maintenance of bacterial RNA transcription are central to chlamydial development, so they must be kept during reversible persistence induced by other stimuli [33]–[37].

We have tried to block the fusion of lysosomes with bacteria using inhibitors of the classical pathways that we suspected to be involved. Our results show that this phenomenon occurs via a pathway insensitive to NH_4_Cl, known to inhibit the fusion of lysosomes with cargo, and that the activation of cathepsin D in pG forms is independent of the activation of a lysosomal H+v-ATPAse (data not shown). Thus, mechanisms involved remain to be elucidated.

The consequences of pG treatment of *Chlamydiaceae in vitro* remain a matter of significant debate in the literature. Our findings are in agreement with reports on the deleterious effect of ß-lactams on *C. trachomatis* or *C. pneumoniae* infections *in vitro* [15], [21], [22]. However, other studies appear in contradiction, in infections by *C. psittacci* [13], [38] or *C. trachomatis* [20], [30]. A detailed inspection of the associated protocols revealed that most authors included CHX in cultures to control cell growth during infection. We have shown that CHX treatment of pG-treated infected cells allows the recovery of an infectious progeny after removal of pG, possibly by inhibiting the process of lysosomes fusion to the bacterial compartment or the apoptosis of infected cells. This observation strongly suggests that the use of CHX is a likely explanation for the different results.

However, other causes might be: a too short incubation time with pG, pG addition outside of its window of action (RB stage), or inadequate pG dosage for the number of infected cells. Each of these conditions would lead to an incomplete bactericidal effect, leaving some bacteria alive, but not to persistence followed by reversion. We have demonstrated that pG acts on RBs of *C. trachomatis* serovar L2 mostly between 12 hpi and 32 hpi, providing important information on the window of action of pG on bacteria. Observations using confocal microscopy show that EBs of *C. trachomatis* serovar L2 differentiate into RBs ∼10 hpi in HeLa cells and that pG causes the formation of pG-forms after only 3-4h of co-incubation with infected cells. If the molecular targets of antibiotics such as tetracycline (targeting 30S ribosomal subunit) and macrolide (targeting the 50S subunit) are well defined in *Chlamydiaceae* and other bacteria, identifying the pG target in the context of the *Chlamydia* paradox is a challenge, as penicillin usually binds to and inhibits penicillin-binding proteins (PBP), involved in peptidoglycan synthesis in other bacteria [12], [39]. Three PBP have been identified in *C. trachomatis* but the biosynthetic pathway of peptidoglycan is incomplete in this bacterium and peptidoglycan has yet to be definitely detected [12], [39]. This suggests that the effect of penicillin on *Chlamydiaceae* might not be related to peptidoglycan synthesis. However, *Chlamydia* could produce a covalently closed, glycan-less polypeptide present in the bacterial wall and whose synthesis is β-lactam sensitive. Its absence could affect the development and the viability of the bacteria by either modifying the structure of the bacterial wall or affecting RB division [12], [40], [41]. Thus, in the presence of β-lactams, the rigidity of the outer membrane of RBs and the regulation of porins may be compromised [42], [43]. This may render the bacteria more sensitive to osmotic and redox stresses and thus explain the abnormal swelling of RBs, and their continued growth along with the inclusion, both phenotypes induced in the presence of pG.

*C. trachomatis* also synthesizes and translocates proteins into the host cell via type III -dependent or -independent secretion systems [44]–[47]. Such factors manipulate host cell pathways including the prevention of fusion of lysosomes with the bacteria. Any structural modification of the bacterial wall could inhibit the secretion of virulence factors and indirectly affect bacterial viability.

Our data show that pG-treatment of *Chlamydia*-infected cells in culture kills the bacteria. We tested this bactericidal effect *in vivo* by infecting mice intra-vaginally by *C. muridarum* and then treating them with doxycycline or pG. In our study, 60% of C57BL/6 females infected by *C. muridarum* developed upper genital tract lesions that can be hydrosalpinx and/or inflammatory cysts along the uterine horns. These observations are consistent with what has been published in recent studies [48]–[49]. We have shown that both antibiotic treatments lead to a faster elimination of *Chlamydia* from the lower genital tract, but only pG treatment significantly protected mice from the development of uterine inflammatory lesions, monitored both macroscopically and microscopically in paraffin sections. In accordance with our study, previous work showed that C57BL/10 mice vaginally-infected with *C. muridarum* and treated by Dox between 10 and 24 days post infection (10–30 days post-infection in our study) eliminated vaginal infection within 5 days [50]. Despite this rapid clearance, more than 25% of those mice showed hydrosalpinx at 55 days post infection, which is in agreement with our results (49±23% of mice presenting tubal lesions 90 days post infection). The difference in percentages of mice with lesions between the two studies could result from the fact that we have included animals presenting uterine cysts, and also that we sacrificed mice 35 days later than in the other study, leaving time for lesions to develop.

Such an effect of molecules from the ß-lactam family had already been reported in other *in vivo* studies, but it could not be explained since β-lactams were claimed to induce the persistence of *Chlamydiaceae in vitro*. These previous studies had shown that β-lactams have a protective activity in models of respiratory and genital infections in mice [51], [52]. Such unexplained protective effect justified the use of β-lactams resistant to penicillinases or a combination of penicillin and β-lactamase inhibitors, which are still considered excellent alternatives to standard treatment against human chlamydiosis. They have been used particularly during pregnancy [53], [54], in PID including acute salpingitis and cervicitis [55]–[63], and in male urethritis [64].

In conclusion, our data provide the basis of a cellular mechanism for the protective effect of ß-lactam antibiotics against chlamydial infection that had been previously observed *in vivo*. These results support the physiological relevance of our findings and their potential therapeutic applications.

## Supporting Information

Figure S1 **The effect of pG on** ***Chlamydiaceae*** **is independent of host cell type, serovar, biovar or species of the bacteria, but is dependent on eukaryotic protein neosynthesis. A-**THP-1, a human monocyte/macrophage tumor cell line, RL95-2, a human endometrial tumor cell line and HeLa, a human cervical tumor cell line were infected by either *C. trachomatis* serovar L2 (*C.t* L2), or serovar D (*C.t* D), or *Chlamydia muridarum*. Infected cells were either treated with pG (100 IU/ml) at 3 hpi or left untreated. Cells were fixed at 24 hpi (RL95-2/*C.t* L2; HeLa/*C.t* D; HeLa/*C. muridarum*) or at 48 hpi (THP-1/*C.t* L2; THP-1/*C.t* D) and stained using Hoechst (blue) and anti-*Chlamydia sp.* antibody (green). Scale bar: 10 µm. The experiment has been repeated four times. **B**-HeLa cells infected by *C. trachomatis serovar* L2 were treated at 3 hpi either with different concentrations of pG (from 0.01 to 100 IU/mL) or left untreated. In some samples (+), pG was removed at 48 hpi from culture medium and cultures were continued for 52 h. At 100 hpi, cellular extracts were processed for the titration of recovered infectious activity. The experiment has been repeated three times. **C-** HeLa cells were treated with cycloheximide (CHX) at 1 µg/ml, 4 h before infection. Cells were then infected by *C. trachomatis serovar* L2 (IFU = 1) and treated with pG (100 IU/ml) at 3 hpi or left untreated. In some experimental conditions, pG was washed away (pG removal) from culture medium at 24 hpi and the culture was continued for 76h. At 100 hpi, cells layers (left panel) and supernatants (right panel) were collected and processed for the titration of recovered infectious activity. The experiment has been repeated three times. *: statistically significant difference (p<0.05), ***: statistically significant difference (p<0.001).
(TIF)

Figure S2 **Cathepsin D is retained in pG-forms of** ***Chlamydia trachomatis*** **independently of biovar and host cells.** RL95-2 and HeLa cells were infected with *C. trachomatis serovar* L2 or *C. trachomatis serovar* D, respectively, and treated with pG at 3 hpi or left untreated. At 24 hpi, cells were fixed and stained with Hoechst, anti-*Chlamydia sp.* antibody and anti-Cathepsin D. The experiment has been repeated three times.
(TIF)

Figure S3 **Uterine horn pathology is significantly decreased in C57Bl/6 mice infected with** ***C.muridarum*** **and treated with pG.** Mice were infected or not with *C.muridarum*, treated or not with antibiotics (Dox or pG) and sacrificed at ninety days after infection (cf. legends to Figure 6). Histopathological evaluation was performed as described in Materials and Methods. All pictures are presented at the same magnification scale (X40). Liquid filled cysts are marked with arrows in untreated infected mice. 1: lumen, 2: uterine epithelium; 3: chorion, 2-3: endometrium; 4-5: myometrium, 4: circular muscle layer, 5: longitudinal muscle layer; 6 serosa; 7: vascular layer between the two muscle layers.
(TIF)

Movie S1 **Time-lapse video showing the recruitment of lysotracker-positive compartments into bacterial inclusions of pG-treated infected cells.** Inclusion (I), Nucleus (N). The experiment has been reproduced four times. Magnification: X 400. Scale bar: 10 µm. (For still images see figure 4D).
(MOV)
