# Supplementary figures and images for: Penicillin Kills Chlamydia following the Fusion of Bacteria with Lysosomes and Prevents Genital Inflammatory Lesions in C. muridarum-Infected Mice
Source: PLoS One. 2013 Dec 23;8(12):e83511. doi: 10.1371/journal.pone.0083511 (PMC3871543; doi:10.1371/journal.pone.0083511)

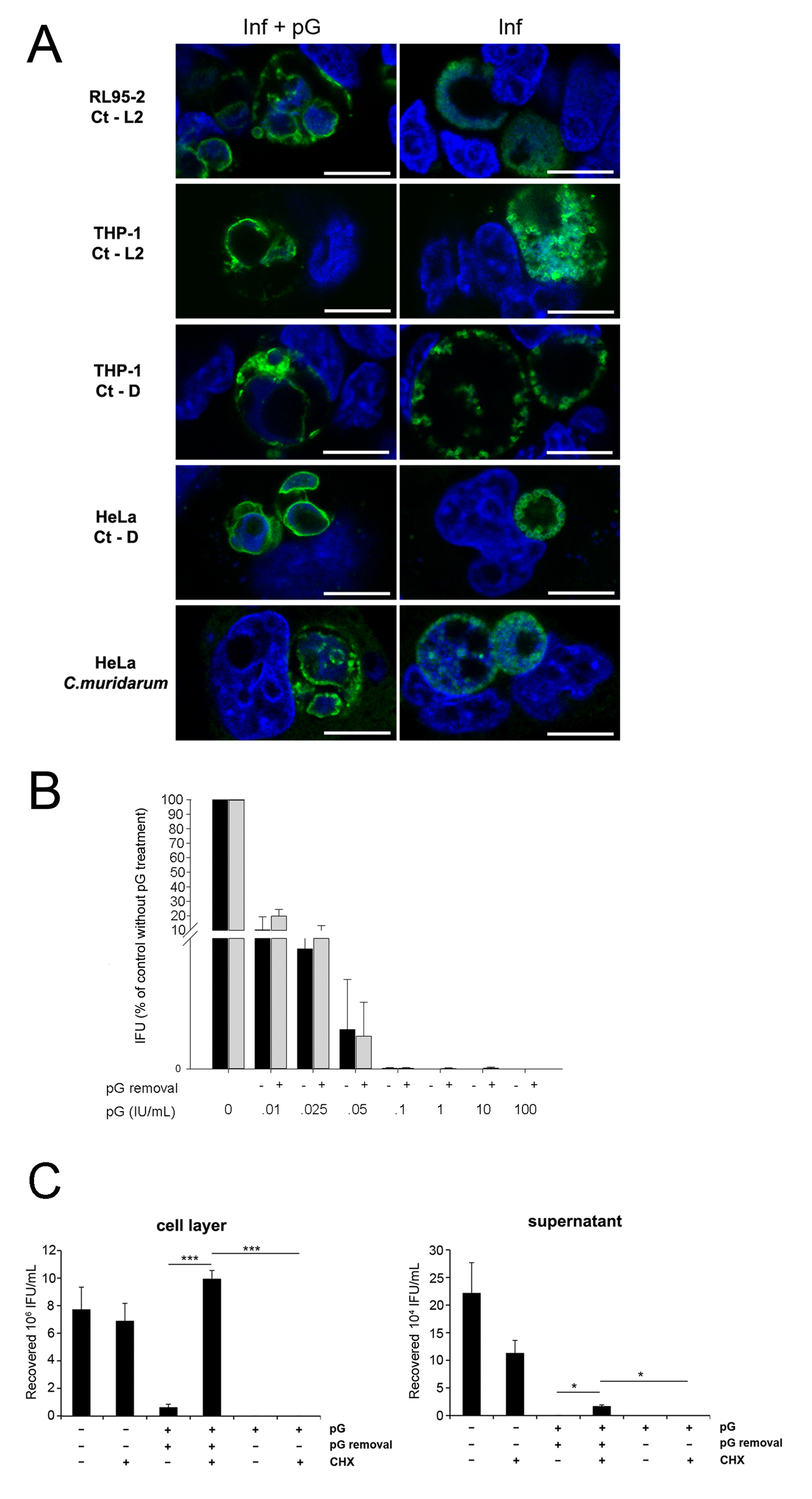

Supplement: Figure S1 — The effect of pG on Chlamydiaceae is independent of host cell type, serovar, biovar or species of the bacteria, but is dependent on eukaryotic protein neosynthesis. A-THP-1, a human monocyte/macrophage tumor cell line, RL95-2, a human endometrial tumor cell line and HeLa, a human cervical tumor cell line were infected by either C. trachomatis serovar L2 (C.t L2), or serovar D (C.t D), or Chlamydia muridarum. Infected cells were either treated with pG (100 IU/ml) at 3 hpi or left untreated. Cells were fixed at 24 hpi (RL95-2/C.t L2; HeLa/C.t D; HeLa/C. muridarum) or at 48 hpi (THP-1/C.t L2; THP-1/C.t D) and stained using Hoechst (blue) and anti-Chlamydia sp. antibody (green). Scale bar: 10 µm. The experiment has been repeated four times. B-HeLa cells infected by C. trachomatis serovar L2 were treated at 3 hpi either with different concentrations of pG (from 0.01 to 100 IU/mL) or left untreated. In some samples (+), pG was removed at 48 hpi from culture medium and cultures were continued for 52 h. At 100 hpi, cellular extracts were processed for the titration of recovered infectious activity. The experiment has been repeated three times. C- HeLa cells were treated with cycloheximide (CHX) at 1 µg/ml, 4 h before infection. Cells were then infected by C. trachomatis serovar L2 (IFU = 1) and treated with pG (100 IU/ml) at 3 hpi or left untreated. In some experimental conditions, pG was washed away (pG removal) from culture medium at 24 hpi and the culture was continued for 76h. At 100 hpi, cells layers (left panel) and supernatants (right panel) were collected and processed for the titration of recovered infectious activity. The experiment has been repeated three times. *: statistically significant difference (p<0.05), ***: statistically significant difference (p<0.001). (TIF) [file pone.0083511.s001.tif]

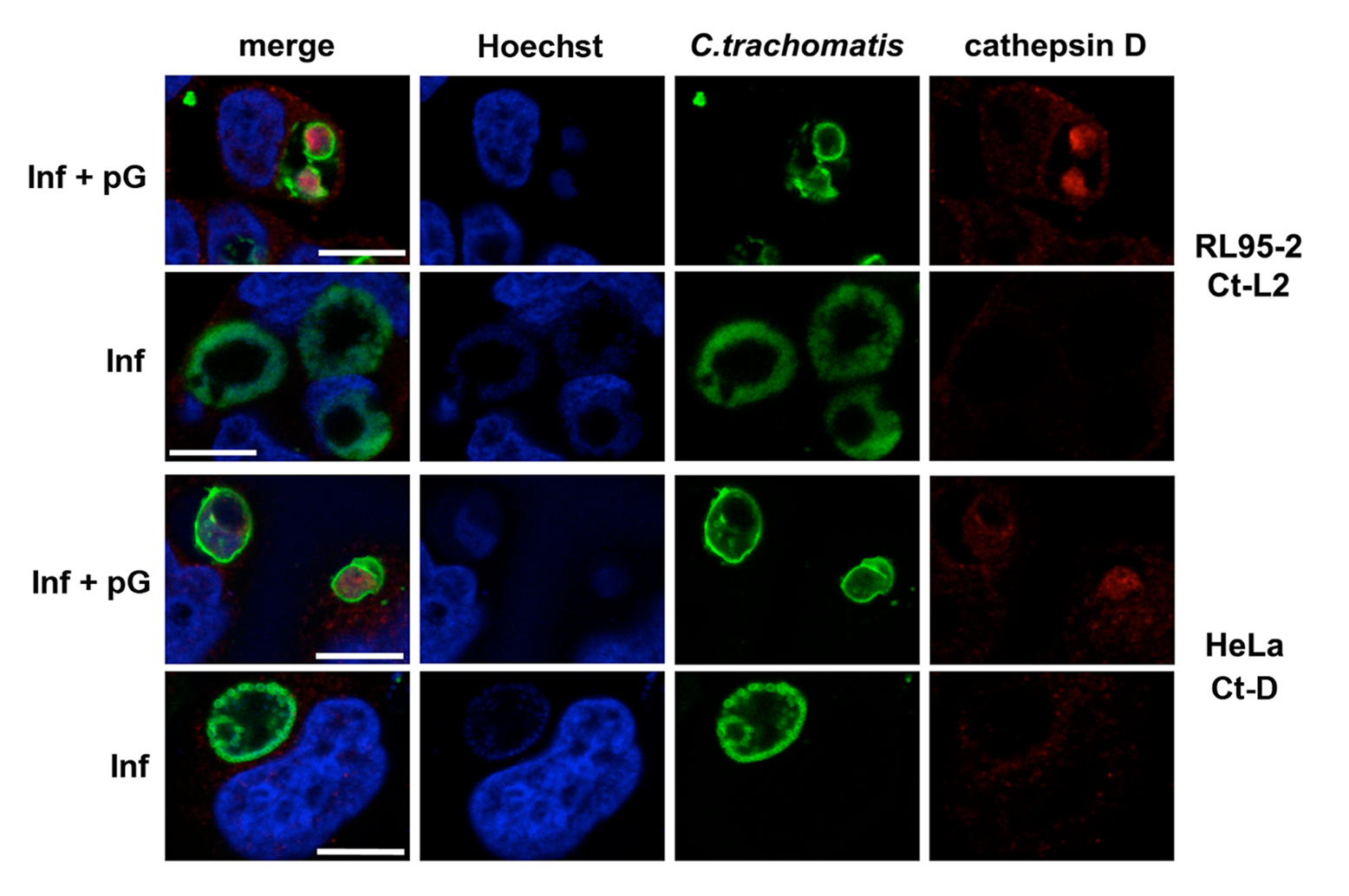

Supplement: Figure S2 — Cathepsin D is retained in pG-forms of Chlamydia trachomatis independently of biovar and host cells. RL95-2 and HeLa cells were infected with C. trachomatis serovar L2 or C. trachomatis serovar D, respectively, and treated with pG at 3 hpi or left untreated. At 24 hpi, cells were fixed and stained with Hoechst, anti-Chlamydia sp. antibody and anti-Cathepsin D. The experiment has been repeated three times. (TIF) [file pone.0083511.s002.tif]

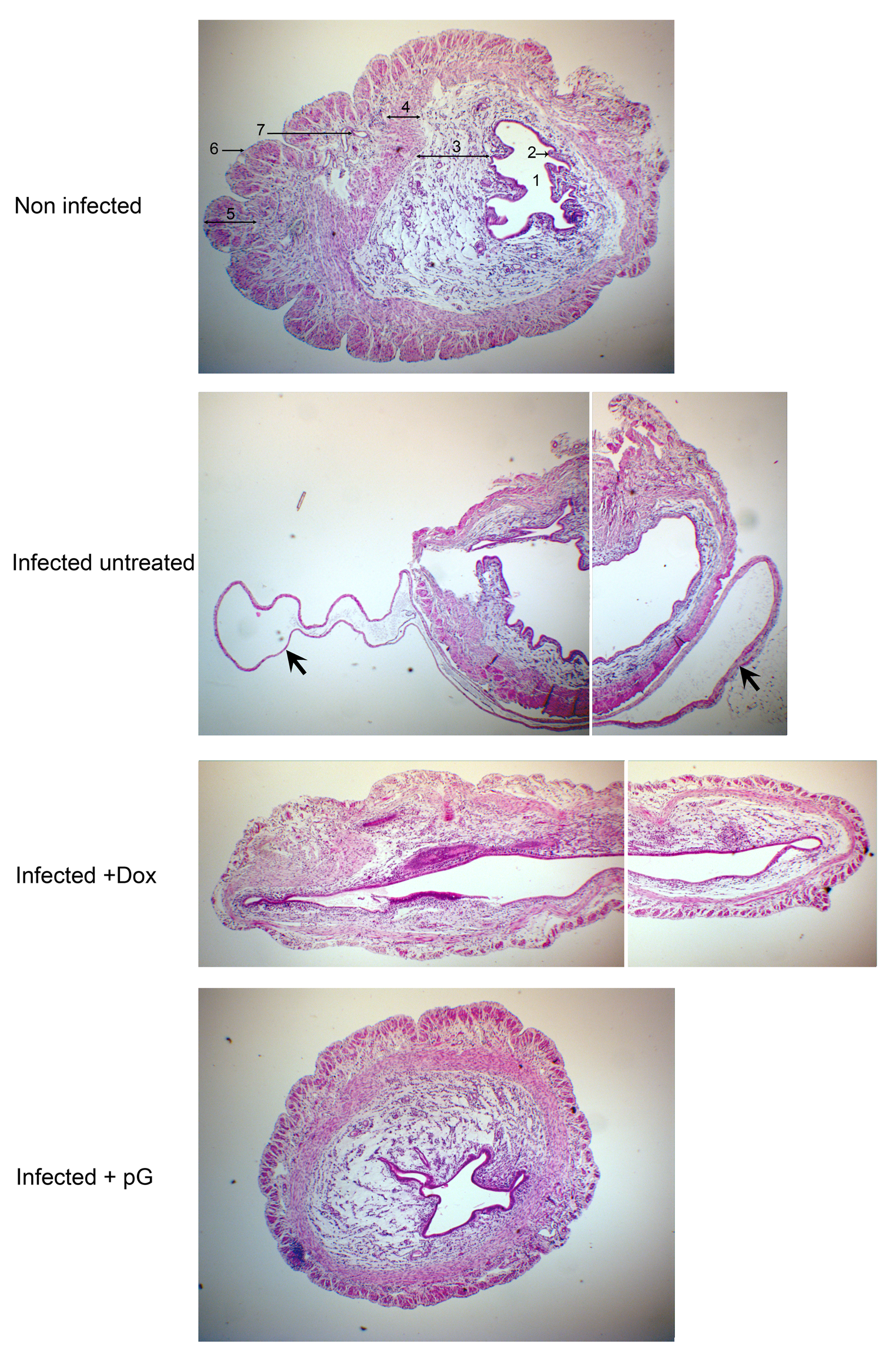

Supplement: Figure S3 — Uterine horn pathology is significantly decreased in C57Bl/6 mice infected with C.muridarum and treated with pG. Mice were infected or not with C.muridarum, treated or not with antibiotics (Dox or pG) and sacrificed at ninety days after infection (cf. legends to Figure 6). Histopathological evaluation was performed as described in Materials and Methods. All pictures are presented at the same magnification scale (X40). Liquid filled cysts are marked with arrows in untreated infected mice. 1: lumen, 2: uterine epithelium; 3: chorion, 2-3: endometrium; 4-5: myometrium, 4: circular muscle layer, 5: longitudinal muscle layer; 6 serosa; 7: vascular layer between the two muscle layers. (TIF) [file pone.0083511.s003.tif]
